# Dynamics of tsetse natural infection rates in the Mouhoun river, Burkina Faso, in relation with environmental factors

**DOI:** 10.3389/fcimb.2013.00047

**Published:** 2013-08-29

**Authors:** Jérémy Bouyer, Naférima Koné, Zakaria Bengaly

**Affiliations:** ^1^Unité Mixte de Recherche Contrôle des Maladies Animales Exotiques et Emergentes, Centre de Coopération Internationale en Recherche Agronomique pour le DéveloppementMontpellier, France; ^2^Unité Mixte de Recherche 1309 Contrôle des Maladies Animales Exotiques et Emergentes, Institut National de la Recherche AgronomiqueMontpellier, France; ^3^Service de Bio Ecologie et Pathologies Parasitaires, Laboratoire National d'Elevage et de Recherches Vétérinaires, Institut Sénégalais de Recherches AgricolesDakar, Sénégal; ^4^Centre International de Recherche-Développement sur l'Elevage en Zone subhumideBobo-Dioulasso, Burkina Faso

**Keywords:** vector competence, vector capacity, environmental stress, parasite extrinsic cycle, infection rate, maturation rate, temperature

## Abstract

In Burkina Faso, the cyclical vectors of African animal trypanosomoses (AAT) are riverine tsetse species, namely *Glossina palpalis gambiensis* Vanderplank (*G.p.g*.) and *Glossina tachinoides* Westwood (*G.*t.) (Diptera: Glossinidae). Experimental work demonstrated that environmental stress can increase the sensitivity of tsetse to trypanosome infection. Seasonal variations of the tsetse infection rates were monitored monthly over 17 months (May 2006–September 2007) in two sites (Douroula and Kadomba). In total, 1423 flies were dissected and the infection of the proboscis, middle intestine and salivary glands was noted. All the positive organs were analyzed using monospecific polymerase chain reaction (PCR) primers. To investigate the role of different environmental factors, fly infection rates were analyzed using generalized linear mixed binomial models using the species, sex, and monthly averages of the maximum, minimum and mean daily temperatures, rainfalls, Land Surface Temperature day (LSTd) and night (LSTn) as fixed effects and the trap position as a random effect. The overall infection rate was 10% from which the predominant species was *T. congolense* (7.6% of the flies), followed by *T. vivax* (2.2% of the flies). The best model (lowest AICc) for the global infection rates was the one with the maximum daily temperature only as fixed effect (*p* < 0.001). For *T. congolense*, the best model was the one with the tsetse species, sex, maximum daily temperature and rainfalls as fixed effect, where the maximum daily temperature was the main effect (*p* < 0.001). The number of *T. vivax* infections was too low to allow the models to converge. The maturation rate of *T. congolense* was very high (94%), and *G. t*. harbored a higher maturation rate (*p* = 0.03). The results are discussed in view of former laboratory studies showing that temperature stress can increase the susceptibility of tsetse to trypanosomes, as well as the possibility to improve AAT risk mapping using satellite images.

## Introduction

In sub-Saharan Africa, African animal trypanosomoses (AAT), transmitted by tsetse flies (genus *Glossina*), are among the main constraints to the development of cattle farming (Itard et al., 2003). Demographic and climatic pressures have modified tsetse habitats, which are more and more fragmented (Van den Bossche et al., 2010). In the Mouhoun river basin, Burkina Faso, the cyclical vectors of AAT are riverine tsetse species, namely *Glossina palpalis gambiensis* Vanderplank (*G.p.g*.) and *Glossina tachinoides* Westwood (*G. t*.) (Diptera: Glossinidae). The fragmentation of tsetse habitats was studied repeatedly in this area, in order to understand its impact on tsetse distribution and densities (Bouyer et al., 2005; Guerrini et al., 2008), population structure and dispersal (Bouyer et al., 2007, 2009; Koné et al., 2011a).

The risk of animal trypanosomoses was also mapped, based on the number of infectious fly per trap per day as a risk indicator (Bouyer et al., 2006; Guerrini and Bouyer, 2007). This risk mapping revealed that the most dangerous river sections were located at the border of protected forests, due to a “border effect” leading to increased tsetse densities. No difference was, however, observed between the tsetse infection rates in the different landscapes and no environmental factor could be associated to this infection rate.

The goal of the present study was to explore the relationship between tsetse infection rate and various environmental factors at a local scale (the Mouhoun river basin).

## Materials and methods

### Entomological survey

**Figure 1:**
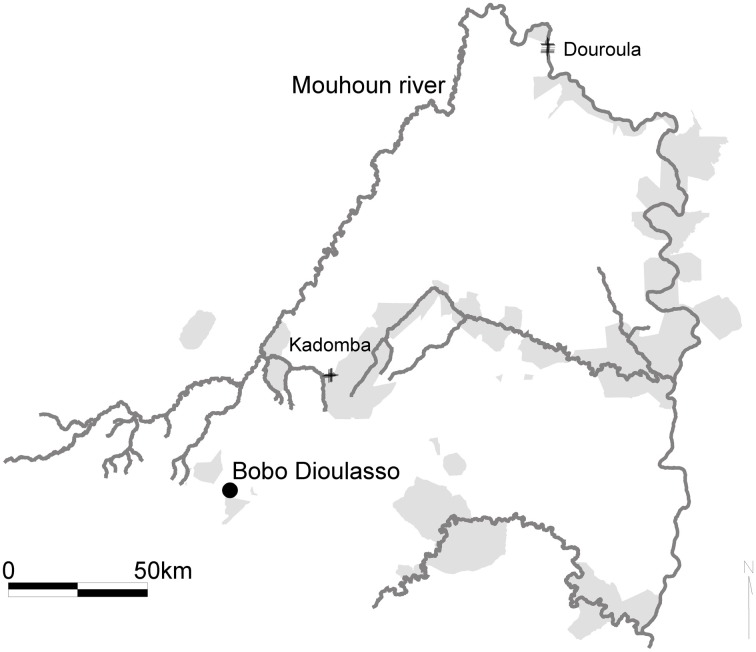
**Location of the study sites in the Mouhoun river basin, Burkina Faso**. The gray areas correspond to the protected forests.

Seasonal variations of the tsetse infection rates were monitored monthly over 17 months (May 2006–September 2007) in two sites, Douroula (12°36′06 ″N, 03°16′54 ″W) and Kadomba (11°32′19″N, 03°58′24 ″W), using 20 and 13 biconical traps, respectively (Figure 1).

These sites were previously characterized regarding vegetation and tsetse population dynamics, and correspond to border of protected forests, with a high risk for AAT transmission, corresponding to the interface AAT cycle (Van den Bossche et al., 2010). Douroula harbors an open Sudanean gallery forest which is the preferred habitat of *G. t*. (85% of the captures) whereas Kadomba harbors a closed Guinean gallery forest which is the preferred habitat of *G.p.g* (99.96% of the captures) (Koné et al., 2011b). The two habitats are comparable in terms of host availability: domestic animals are largely predominant, particularly cattle and small ruminants. Intensive poaching has destroyed all wild mammals, with the exception of *Tragelaphus scriptus* and *Cephalophus rufilatus*, present in very low densities (Bouyer et al., 2005). Reptiles are still abundant, and represented mainly by *Varanus niloticus* and *Crocodylus niloticus*. In total, 1423 flies were dissected (94 *G.p.g* and 534 *G. t*. in Douroula, and 795 *G.p.g* in Kadomba), and the infection of the proboscis, salivary glands and middle intestine was noted (the organs were dissected in this order). When at least one of the organs was positive, all the three organs of a given tsetse were analyzed using monospecific polymerase chain reaction (PCR) primers for *Trypanosoma brucei* sensu lato, *T. congolense* savannah type only and *T. vivax* (Solano et al., 1999; Desquesnes and Davila, 2002). For *T. congolense*, flies presenting an infected proboscis were considered as mature infections.

### Environmental data

The maximum, minimum and mean daily temperatures and rainfalls were obtained from meteorological stations located at 25 and 50 km from the rivers sections monitored in Douroula and Kadomba, respectively.

In addition, daily and nightly Land Surface Temperatures (LSTd and LSTn, respectively) measured from MODIS satellites were used as monthly average of the values in the 1^*^1 km pixels intersecting the sites where traps were set.

### Statistical analyses

The fly infection rates were analyzed using generalized linear mixed binomial models (Laird and Ware, 1982) using the species, sex, and monthly averages of the maximum, minimum and mean daily temperatures, rainfalls, LSTd and LSTn as fixed effects and the trap position as a random effect. The best model was considered as the one with the lowest corrected Akaike information criterion (AICc) (Hurvich and Tsai, 1995; Burnham and Anderson, 2002).

The R software (R Core Team, 2013) was used for statistical analysis, together with the lme4 package for the linear mixed-effect model (Bates et al., 2011) and the MuMin package for the implementation of the AICc (Burnham and Anderson, 2002).

## Results

### Infection rates

The overall infection rate was 10% from which the predominant species was *T. congolense* (7.6% of the flies), followed by *T. vivax* (2.2% of the flies). For the remaining infected flies, the trypanosome species could not be identified and *T. brucei* was not observed.

**Figure 2:**
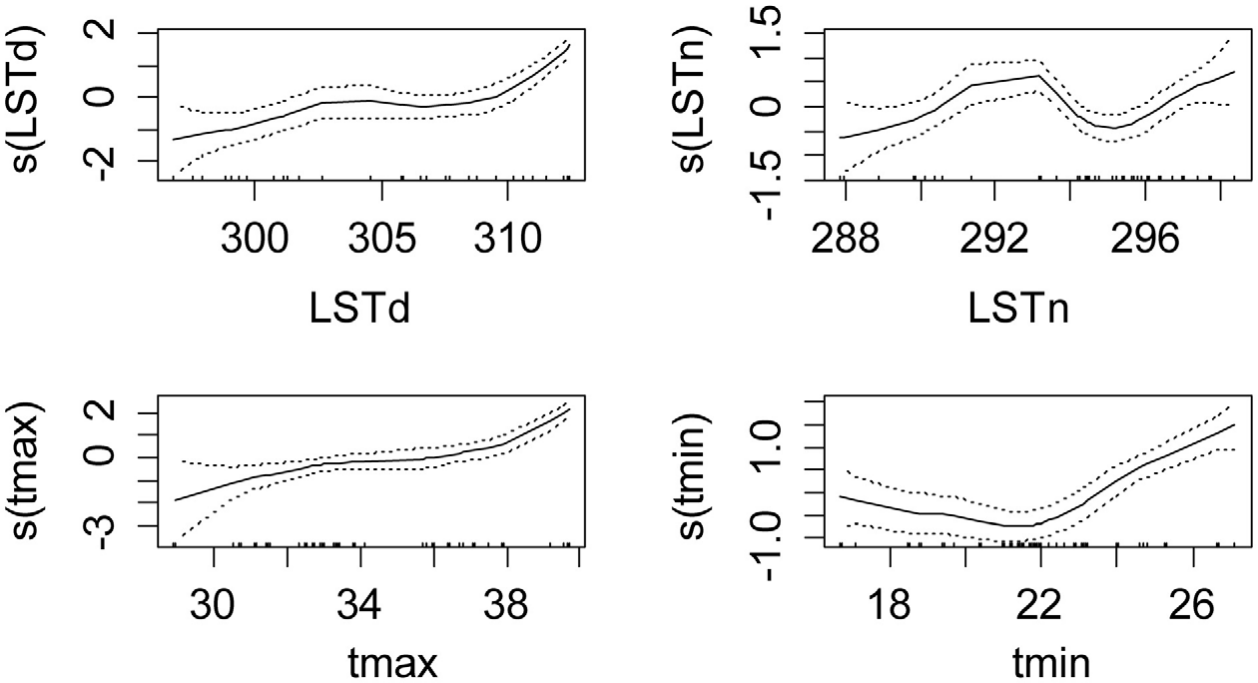
**Shape and amplitude of the relationships between tsetse infection rate and environmental variables**. The values were estimated by a logistic regression model of cubic spline functions of environmental variables (LSTd and LSTn: MODIS Land Surface Temperature day and night in kelvins, tmin and tmax: minimum and maximum monthly averages of air temperature in °C measured in meteorological stations). For each environmental variable y, s(y) represents the fit of the regression model and the uncertainty lines present the standard error.

The best model for the global infection rates was the one with the maximum daily temperature only as fixed effect (*p* < 0.001). The sex, age and other environmental variables did not improve the model predictions. The plot of cubic spline functions of environmental variables shows that the best relationship is actually observed for the maximum air temperature (Figure 2). LSTd had a similar relationship, although the reduction of the extent illustrates the weaker relationship. The increased confidence rate at low mean temperatures probably illustrated the intervention of other factors, like nutritional stress or others. LSTn and the minimum temperature did not present linear relationship with the infection rate.

**Figure 3:**
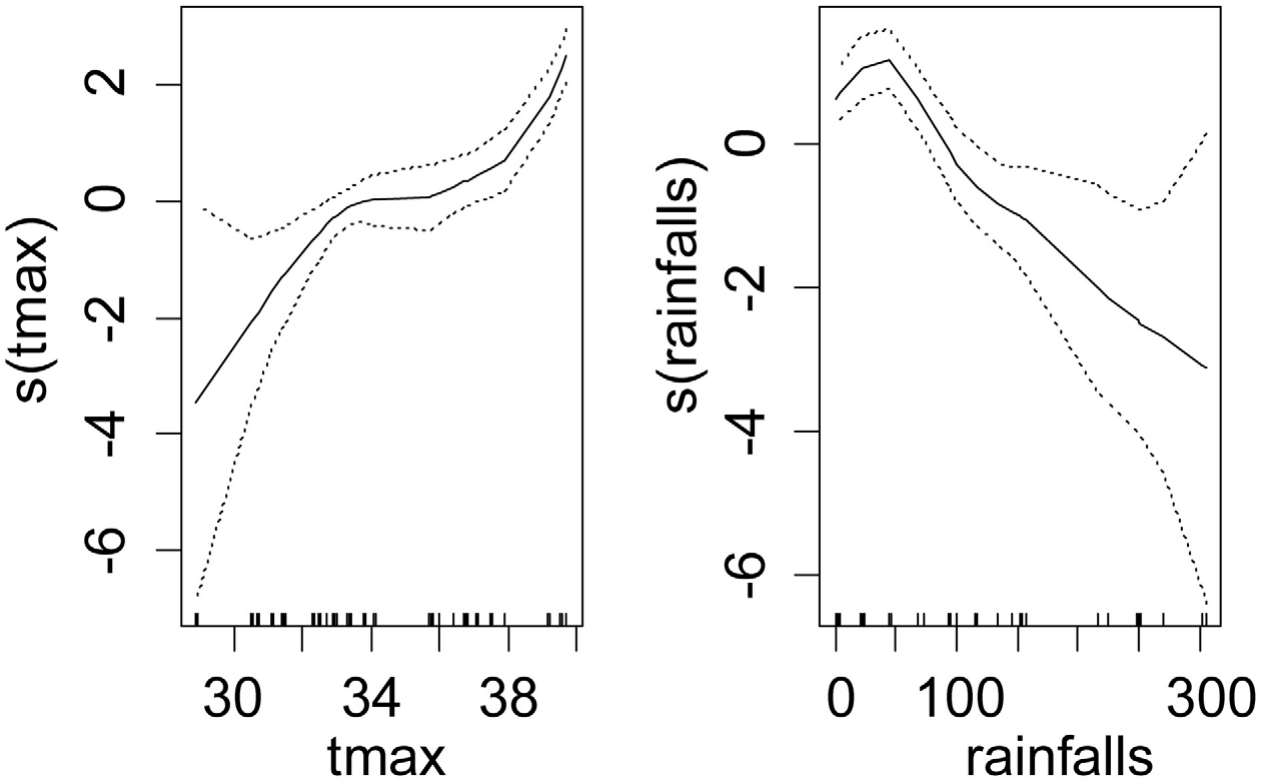
**Shape and amplitude of the relationships between tsetse infection rate with *T. congolense* and environmental variables**. The values were estimated by a logistic regression model of cubic spline functions of environmental variables (tmax: maximum monthly averages of air temperature in °C measured in meteorological stations, rainfalls: total monthly rainfalls in mm). For each environmental variable y, s(y) represents the fit of the regression model and the uncertainty lines present the standard error.

For *T. congolense*, the best model was the one with the tsetse species, sex, maximum daily temperature and rainfalls as fixed effect, where the maximum daily temperature was the main effect (*p* < 0.001) and the sex and rainfalls had marginal effects on the infection rate (negative for females and for rainfalls). Table 1 presents the fixed coefficients of the best model. A similar but stronger relationship was observed between the maximum air temperature and the infection rate (Figure 3). Monthly rainfalls had a weaker inverse relationship with the infection rate.

**Table 1:** **Fixed effects of the best prediction model for *T. congolense* infection rates**.

**Parameter**	**Estimate**	**Std. Error**	***z*-value**	**Pr(>|z|)**
(Intercept)	−15.32	1.98	−7.75	9.09e-15^***^
*G. tachinoides*	0.30	0.24	1.25	0.21
Female	−0.39	0.22	−1.80	0.0712·
tmax	0.36	0.05	6.84	8.12e-12·
Rainfalls	−0.005	0.002	−1.96	0.0501·

Definition of the parameters: tmax, maximum monthly averages of air temperature in °C measured in meteorological stations; rainfalls: total monthly rainfalls in mm.

Significance codes: 0^***^; 0.05·.

The number of *T. vivax* infections was too low to allow the models to converge.

### Maturation rates

The maturation rate of *T. congolense* was very high (94%). The best model was the one with tsetse species, sex and maximum daily temperature as fixed effects, and only the species effect was significant, *G. tachinoides* having a higher maturation rate (99%, s.d. 1%) than *G. p. gambiensis* (92%, s.d. 5%, *p* = 0.03).

## Discussion

Trypanosomosis is also an infection for tsetse, which in turn try to control it through pre-existing defenses and a immune gene expression that is induced upon feeding on trypanosomes (Dyer et al., 2013 # 1145). Many studies of vector manipulation by parasites demonstrated that parasites can impact vector survival, trophic behavior, fecundity (Lefevre and Thomas, 2008). Studies conducted in experimental conditions revealed that nutritional stress can increase their susceptibility to infection by trypanosomes (Akoda, 2009). For example, starvation increased the infection rates of *Glossina morsitans morsitans* Westwood with *Trypanosoma congolense* and the maturation rate of *T. brucei brucei* infections (Kubi et al., 2006). It was thereafter demonstrated that fly starvation can lead to a decreased expression of immune genes in newly hatched flies or a lack of immune responsiveness to trypanosomes in older flies, that can contribute to increased susceptibility of nutritionally stressed tsetse flies to trypanosome infection (Akoda et al., 2009b). In the case of tsetse infections with *Trypanosoma brucei brucei*, nutritional stress can also result in enhanced maturation of the trypanosome infection (Akoda et al., 2009a). It was also demonstrated that exposure of adult flies to high temperatures can increase the development rate of trypanosomes in adult flies (Leak, 1998). Moreover, flies emerging from pupae exposed to high temperatures are also more sensitive to infections by *T. b. rhodesiense* (Burtt, 1946) and *T. congolense* (Ndegwa et al., 1992). The impact of heat stress on tsetse immunity might thus be one of the explanations for the pattern observed in this study.

Under field conditions, a positive correlation was also observed between latitude, associated to increasing mean annual temperature and tsetse infection rates (Ford and Leggate, 1961). The authors stated that “at higher altitudes or, as in Zululand, a situation approaching the temperate zone, low infections occur as a response to cooler climates.” In that study, the regression of proboscis-type infections on mean annual temperature was much more marked than that of gut and/or gut and proboscis-type infections, which is surprising given that a decreased immune response associated to heat stress is probably involved.

In our study, we observed a similar pattern at a more local scale and between seasons, i.e., a strong increase of infection rates with mean monthly maximum temperature. Moreover, the regression of all infections together on mean monthly temperature was less marked than for that of gut and/or gut and proboscis-type infections (*T. congolense*). Since we used monthly average temperatures, both exposure of the pupae and the adult flies could have contributed to the observed pattern. Cattle rearing systems and farmer habits have been characterized in the study sites (Koné et al., 2012), together with a monitoring of AAT prevalence in sentinel herds (Métras et al., 2008). The sociological study showed that herders, particularly those of the Fulani ethnic group, tend to avoid the main river during the rainy season, when temporary ponds are available. Tsetse are, however, able to disperse outside the gallery forest during this season, up to 2km in the neighboring savannah (Cuisance et al., 1985; Vreysen et al., 2013). Overall, the AAT infection rates in cattle were similar between sites but showed a significant peak in July (middle of the rainy season), which is different from the peak of infection observed in tsetse (hot dry season, from April to May, when temperatures were highest). It is thus not likely that the pattern observed in this study was driven by an increased contact with infected hosts and this indirect effect would not give such a strong signal.

The impact of temperature on the maturation rate of *T. congolense* was not significant, which might be due to overall very high maturation rates observed. This maturation rate might have been overestimated by considering any fly positive in its proboscis as a mature infection.

Mapping vectorial diseases rely on mapping different risk indicators, like the vectorial capacity (Tran et al., 2005). The main elements of the vectorial capacity are the relative density of vectors to hosts, the survival of vectors, their feeding preferences and their infectious rate. Recent AAT risk modeling efforts based on satellite images in the Mouhoun river mainly relied on mapping tsetse catches per trap per day, which are considered to be correlated to their relative densities to the hosts (Bouyer et al., 2006; Guerrini and Bouyer, 2007). Even if the number of infectious fly per trap per day were considered, corresponding to the entomological inoculation rate, an indicator commonly used to map malaria risk for example, no environmental predictor of tsetse infection rate was evidenced. The integration of the results presented here in these AAT mapping efforts would probably lead to improved accuracies of the maps, and since the densities of tsetse are relatively stable over time (k demographic strategy) (Koné et al., 2011b), it might also allow making seasonal predictions. Of course, mechanical transmission cannot be neglected (Desquesnes et al., 2009) and can lead to a risk of trypanosomosis independent from tsetse, including outside the tsetse belt (Pagabeleguem et al., 2012). Moreover, high temperature might have contradictory impacts on different factors of transmission, for example by reducing tsetse lifespan and density (Van den Bossche et al., 2010).

In our study, it was surprising that air temperature measured from meteorological stations located quite far from the study sites better predicted tsetse infection rates than the MODIS LSTd measured in the 1^*^1 km pixels where the tsetse were captured. This is probably due to discrepancies between LSTd and air temperature, in relation to land cover, whereas the maximum air temperatures in the meteorological stations were probably highly correlated to those of the study sites. It would be very interesting to conduct a similar study including the monitoring of air temperature and hygrometry using field meteorological stations. In our study, the correlation between LSTd and max temperature was very high (*r*^2^ = 0.77, *p* < 10^−3^). LSTd was still highly correlated to the infection rate, even if less powerful than max temperature to predict it. LSTd is a MODIS composite synthetic variable. In Senegal, we, however, observed important discrepancies between air temperature measured on the ground and LSTd. Other indexes like NDVI and MIR could be combined to the latter to better predict air temperature, with an accuracy of ~1°c. Actually, these parameters are correlated to land cover, which in turn impacts air temperature. Efforts are presently underway in Senegal to build models of air temperatures using a network of field meteorological stations. The inverse relationship observed with monthly rainfalls in the case of *T. congolense* might be due to a mitigation of the evapotranspiration caused by high temperatures by an increase of relative hygrometry. Actually, tsetse survive longer at high temperatures when relative hygrometry is higher (Buxton, 1955).

A recent model predicted a large-range expansion of sleeping sickness in East and South Africa, caused mainly by a shift of up to 60 per cent of its geographical extent (Moore et al., 2012). This change was mainly anticipated from predicted changes in global temperatures impacting on the following entomological and epidemiological parameters: total tsetse population size, natural death rate of tsetse, parasite maturation rate in tsetse and tsetse biting rate. However, the potential impact of temperature on tsetse primary infection rate was not considered and might improve greatly model predictions. The raw data generated in this survey are made available to encourage modeling efforts (Supplementary file 1). Trap locations can also be requested from the corresponding author.

### Conflict of interest statement

The authors declare that the research was conducted in the absence of any commercial or financial relationships that could be construed as a potential conflict of interest.
